# Health equity in Nebraska: addressing disparities through place-based policy innovation

**DOI:** 10.3389/fpubh.2025.1651092

**Published:** 2025-09-17

**Authors:** Changmin Yan, Michelle L. Hughes, Lindsey B. Crawford, Maria Cantu-Hines, Roopan Miriam George, Dipti A. Dev, Gregory R. Bashford

**Affiliations:** ^1^College of Journalism and Mass Communications, University of Nebraska-Lincoln, Lincoln, NE, United States; ^2^Department of Special Education and Communication Disorders, University of Nebraska-Lincoln, Lincoln, NE, United States; ^3^Department of Biochemistry, University of Nebraska-Lincoln, Lincoln, NE, United States; ^4^Four Corners Health Department, York, NE, United States; ^5^Department of Nutrition and Health Sciences, University of Nebraska-Lincoln, Lincoln, NE, United States; ^6^Department of Child, Youth and Family Studies, University of Nebraska-Lincoln, Lincoln, NE, United States; ^7^Department of Biological Systems Engineering, University of Nebraska-Lincoln, Lincoln, NE, United States

**Keywords:** health disparities, social determinants of health, rural health, health equity, public health policy, mental health services, Nebraska health

## Abstract

This policy paper examines persistent health disparities in Nebraska, drawing on insights from a symposium that engaged more than 180 health professionals and organizational leaders statewide. Nebraska’s dual burden of rural provider shortages and urban socioeconomic inequities highlights the need for place-based policy solutions tailored to diverse regional drivers of health disparities—an approach that can be adapted by other states facing similar urban–rural divides. The paper urges Nebraska-focused public health policies with strategies other states can adapt. Key recommendations include expanding pediatric mental health services, implementing community-based interventions targeting social determinants of health, and increasing access to care through telehealth, and culturally and linguistically appropriate services. These approaches aim to advance equity in Nebraska and inform broader national efforts to reduce health disparities.

## 1 Introduction

Health disparities are preventable differences in health outcomes that are closely linked with social, economic, and environmental disadvantage (1). These disparities disproportionately affect low-income individuals, people of color, LGBTQ+ populations, and those in rural or under-resourced areas, driven by factors such as discrimination, language barriers, and limited access to care, education, and nutritious food. Health disparities accounted for approximately $93 billion in additional medical expenses and up to $42 billion in reduced productivity resulting from early mortality in 2023 in the United States (2). Nebraska, a Midwestern state with both urban and rural populations, presents a distinctive combination of health disparity challenges. While its urban–rural mix is typical of the Midwest, Nebraska’s particular configuration, i.e., long distances between population centers, significant provider shortages, and geographically isolated communities, intensifies access issues. For example, compared to Iowa, Nebraska’s rural residents often travel farther to reach primary or specialty care due to more sparsely distributed infrastructure. Unlike Kansas or the Dakotas, Nebraska’s urban areas such as Omaha and Lincoln also feature dense pockets of racial and ethnic diversity, requiring culturally responsive services that are less emphasized in neighboring states with more homogenous urban populations. In some ways, this combination parallels patterns seen in Western states like Oregon and Alaska, where a similar rural–urban contrast presents unique logistical and equity challenges in delivering care. These intersecting geographic and demographic dynamics contribute to persistent disparities in healthcare access, especially for pediatric and behavioral health services.

**Figure 1:**
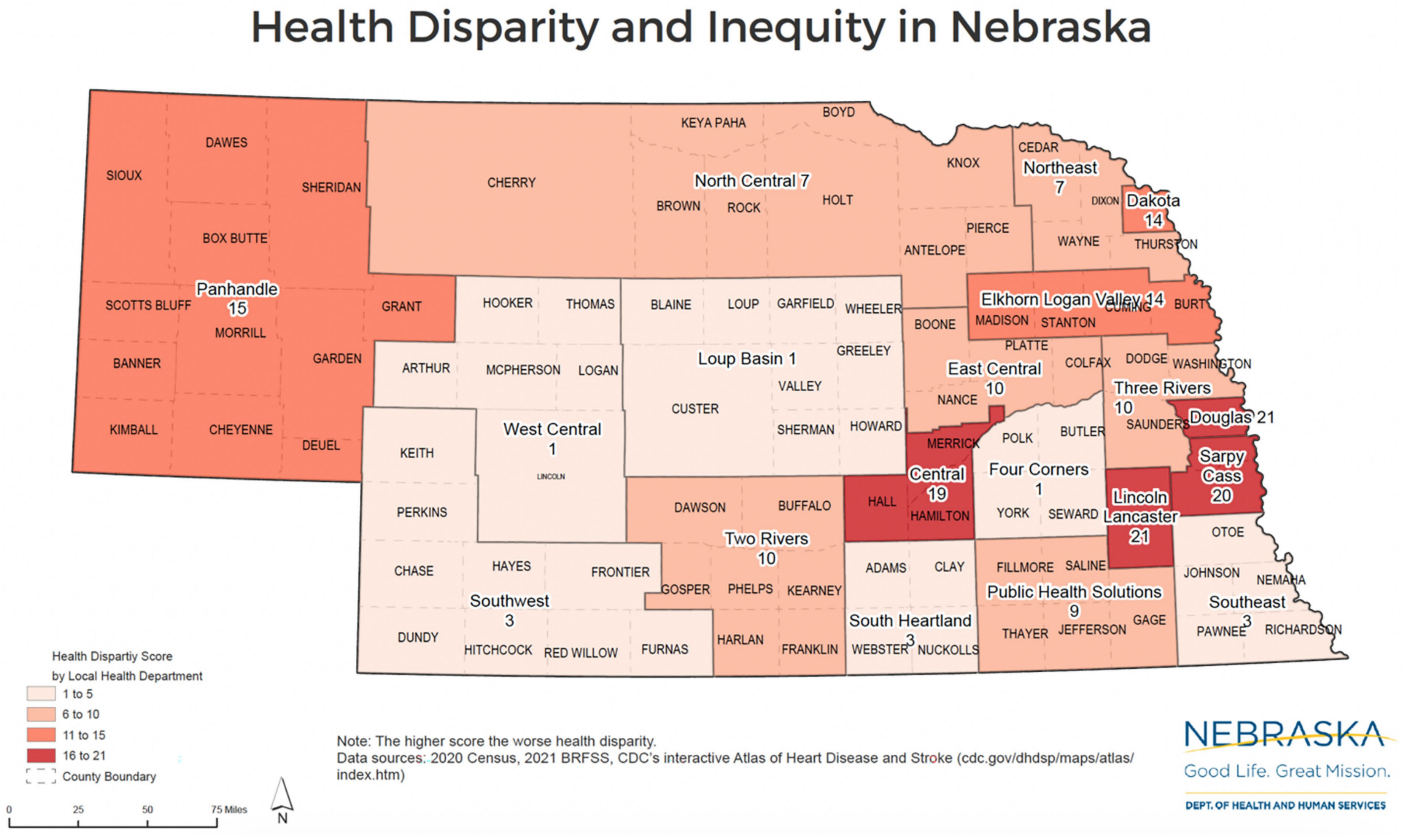
Nebraska’s health disparity map.

Health disparities in Nebraska are not confined to either rural or urban settings; rather, both contain pockets of significant inequity that stem from distinct demographic and structural challenges. As illustrated in the Nebraska Department of Health and Human Services’ *Health Equity Map* (Figure 1), high health disparity scores appear in counties across the entire state, including densely populated urban areas like Douglas and Lancaster counties, as well as remote rural regions such as Scotts Bluff in the Panhandle, and Hall, Hamilton, and Merrick counties, members of the Central District Health Department. While urban counties often struggle with issues related to concentrated poverty, racial and ethnic diversity, and access to culturally competent care, rural counties face challenges like provider shortages, transportation barriers, and limited healthcare infrastructure. This dual burden highlights the need for differentiated, place-based policy solutions tailored to the specific drivers of inequity in each region.

This paper is guided by Bronfenbrenner’s Social Ecological Model (1979), which conceptualizes health outcomes as the result of dynamic interactions across multiple levels of influence, i.e., individual, interpersonal, community, and policy (3). The model has been widely adapted in public health to design multi-level interventions that address both proximal and structural determinants of health (4, 5). By situating our policy recommendations within this framework, we highlight how macro-level policies can shape community structures, interpersonal networks, and individual behaviors, ultimately supporting comprehensive and sustainable strategies for advancing health equity.

Moreover, these challenges reflect broad health disparities seen across the midwestern regions of the United States, where rural areas often suffer from a lack of healthcare access and urban areas face different socioeconomic pressures (6). To address these critical issues, the 2024 “Understanding Health Disparities in Nebraska” symposium brought together experts from various fields, including community health, behavioral health, public health equity, pediatric research, nutrition education, and rural health. The event was promoted through a statewide recruitment campaign that included emails to all local and district health departments, federally qualified health centers across Nebraska, and tribal health organizations serving the Omaha, Winnebago, and Ponca Tribes. Outreach was also extended to contacts affiliated with Nebraska’s four major universities and community organizations involved in public and behavioral health. Additional promotion was conducted via LinkedIn, targeting a broad statewide network of healthcare providers, public health professionals, and academic institutions. Attendees included representatives from diverse geographic and demographic communities, including Native tribes in Nebraska. The Nebraska Department of Health and Human Services Office of Health Disparities and Health Equity, featuring a Native American liaison and district offices covering all three congressional districts, was also represented and contributed as a guest speaker. In total, the symposium engaged 70 in-person attendees and 112 virtual participants, representing organizations such as healthcare providers, community organizations, academic institutions, and public health departments. These experts provided a comprehensive understanding of the multifaceted nature of health disparities in Nebraska. Their insights highlighted the need for targeted public health policies and interventions to promote equity and improve health outcomes (7). This brief is designed to be accessible to both policymakers and non-specialist audiences, with technical concepts explained in plain language to support broad understanding and practical application.

## 2 Evidence sources

The policy recommendations in this paper are informed by a synthesis of peer-reviewed research, publicly available health data, and state and federal reports, as well as presentations and discussions from the 2024 *Understanding Health Disparities in Nebraska* symposium. Sources were identified through targeted searches of databases such as PubMed and Google Scholar, Nebraska Department of Health and Human Services publications, County Health Rankings & Roadmaps, and national health surveillance systems (e.g., CDC WONDER, U. S. Census Bureau). Inclusion prioritized materials that (1) addressed Nebraska-specific health disparities or comparable geographical contexts, (2) provided quantitative or qualitative data relevant to the policy areas under review, and (3) came from credible governmental, academic, or professional organizations. Gray literature, such as policy briefs and technical reports, was included when produced by recognized health policy or public health authorities. Presentations from symposium experts, including community health practitioners, behavioral health providers, public health equity leaders, pediatric researchers, nutrition educators, and rural health specialists, were evaluated for their relevance, alignment with existing evidence, and applicability to Nebraska’s context. All evidence, whether from published literature or expert presentations, was assessed for relevance, timeliness, and applicability to Nebraska, with emphasis on data and insights that could inform actionable, scalable policy strategies.

While the policy recommendations in this brief are grounded in peer-reviewed literature, publicly available datasets, and insights from subject matter experts, they are based primarily on observational evidence and synthesis of prior work. As such, causal relationships should be interpreted with caution, as unmeasured confounding factors and contextual differences across communities may influence outcomes. These recommendations should be considered as evidence-informed strategies, with their effectiveness requiring ongoing evaluation in real-world settings.

## 3 Health disparity policy at the state level

Health disparity policy needs to be driven at the state level due to the unique geographic, demographic, and socioeconomic challenges each state faces. State-specific policies allow for tailored approaches that address local conditions and needs. In addition, state-level collaborations between public health departments and various organizations are increasingly recognized as essential for promoting positive public health outcomes and reducing health inequalities. These collaborations allow for the development and implementation of policies that are finely tuned to the specific challenges of different regions within a state (8). For instance, the National Conference of State Legislatures highlights the importance of state-specific health policies in addressing rural health disparities (9). Nebraska, with its mix of urban and rural populations, exemplifies the diverse challenges faced by midwestern states in the U. S.

### 3.1 Geographic differences

Rural areas in Nebraska face persistent healthcare challenges, including a shortage of providers, particularly in specialty areas of medicine and allied health. Fourteen counties lack a single primary care physician and 61.5% of the state’s Health Professional Shortage Areas are located in rural regions (10). Many rural counties also do not have access to essential specialists, including obstetricians/gynecologists, mental health providers, and oncologists, creating gaps in care for maternal health, cancer treatment, and behavioral health services (11, 12). As a result, rural residents are often forced to travel long distances to urban centers or forgo needed care entirely. Patients in these areas often travel 30 to 60 miles or more to access specialty care. In addition, limited broadband access hinders the effective use of telehealth as nearly 22% of rural Nebraskans lack high-speed internet service (13) and have limited healthcare infrastructure. Childcare availability is another critical factor shaping health and economic stability, particularly in rural areas where the number of licensed childcare providers is often limited, and costs can exceed the state average. According to County Health Rankings and Roadmaps (2025), these disparities are most pronounced in counties with high poverty rates and limited economic opportunities (14). In Nebraska, 10 out of 70 reporting counties have less than five childcare centers per 1,000 children. These issues are common in rural areas across the U. S., but their specific manifestations vary by state, making state-driven policies essential for tailoring solutions to local infrastructure, population needs, and resource availability (6, 12, 15).

### 3.2 Demographic differences

Nebraska’s demographic diversity is concentrated in its urban centers. Omaha (pop. ~483,000) is 68.8% White, 11.5% Black, 4.3% Asian, and 12.9% Hispanic or Latino, while Lincoln (pop. ~294,000) is 77.2% White, 4.2% Black, 4.5% Asian, and 8.8% Hispanic or Latino (16). In contrast, rural areas, home to 28.1% of the state’s population, are 86.0% White (17). This urban concentration of racial and ethnic diversity calls for culturally responsive health services.

In Omaha, neighborhoods like North and South Omaha face high poverty and food insecurity rates, reaching up to 48.5% in some areas (18). These communities, largely composed of racial and ethnic minorities, also experience elevated rates of chronic illness, underinsurance, and mistrust of healthcare systems (19). In Lincoln, immigrant and refugee groups, including Yazidi, Vietnamese, and Sudanese populations, face language and health literacy barriers that contribute to unmet care needs (20). In addition, LGBTQ+ youth across the state face acute mental health disparities, with 73% reporting anxiety and 50% seriously considering suicide, especially among transgender and nonbinary youth (21).

These disparities are not only the result of current gaps in culturally responsive services, but also reflect long-standing structural inequities reinforced by past and existing policies. For instance, redlining and discriminatory housing practices historically restricted where communities of color could live, limiting access to high-quality schools, healthcare, and jobs; effects that are still visible in areas of Omaha and Lincoln today (22, 23). In North Omaha, for example, formerly redlined neighborhoods continue to face disinvestment and concentrated poverty, contributing to poorer health outcomes (18). Addressing demographic disparities therefore requires not only the implementation of new equitable policies, but also a critical review of existing systems and legacy policies that may continue to perpetuate inequity.

### 3.3 Socioeconomic differences

Socioeconomic status, which includes income, education, employment, and housing stability, greatly influences health outcomes. In Nebraska, socioeconomic disparities are evident in both rural and urban settings (24). For example, in North Omaha, a historically underserved urban area, the poverty rate exceeds 25% compared to the state average of around 11% (25). In many rural counties, over 15% of residents live below the poverty line, and high school graduation rates are significantly lower than the state average of 89% (26). The two root causes of poverty and lower educational attainment are compounded by structural barriers to participation in educational opportunities, job training programs, and safety net services. Many of these programs have eligibility criteria based on income thresholds, employment status, or residency requirements, which can exclude residents who still face substantial need. In rural areas, participation is further hindered by infrastructure gaps and broadband access—nearly 22% of rural Nebraskans lack high-speed internet service (13)—as well as the costs of devices, transportation, or program fees. These same limitations inhibit telehealth offerings, reducing access to remote education, job training, and healthcare services (discussed further in the *Healthcare Access for Underserved Populations* section). Pairing addressing structural barriers with supportive services like financial counseling, when designed to complement fair-wage and benefits policies, can help families navigate resources without placing additional burdens on their time or income.

Addressing these root causes requires targeted policy actions that complement broader health equity efforts. State policies, such as those expanding access to affordable housing, improving educational opportunities, increasing job training programs, and strengthening safety net services, can be tailored to address these socioeconomic factors, promoting health equity (8, 16). More specifically, expanding affordable early childhood education, providing wraparound academic and social supports in K–12 schools to improve graduation rates, and investing in workforce development and job creation initiatives in high-poverty areas are critical steps. These strategies should be implemented alongside efforts to expand broadband access, reduce participation costs, and improve infrastructure in underserved rural communities, thereby increasing access not only to education and job training but also to telehealth and other essential services.

Importantly, a review of current and historical policies is needed to understand how systemic barriers, such as redlining, discriminatory lending, and unequal infrastructure investment, have contributed to present-day socioeconomic disparities (22, 23). These policies have created generational wealth gaps and led to under-resourced communities. In Nebraska, these effects are particularly evident in urban neighborhoods like North and South Omaha, as well as rural communities historically excluded from broadband access, quality housing, and healthcare infrastructure (13, 18). Addressing these root causes is essential for advancing meaningful and sustainable health equity.

Addressing Nebraska’s health disparities requires more than a general approach; it demands targeted, state-specific strategies that leverage local expertise and resources. The following sections outline concrete policy options and evidence-based interventions across key focus areas, including pediatric mental health, social determinants of health, and access to care, that together form a blueprint for advancing health equity. These strategies not only respond to Nebraska’s unique needs but also offer scalable solutions that other states with similar profiles can adapt.

## 4 Pediatric mental health services

### 4.1 Current situation

Nebraska faces concerning trends in youth mental health that exceed national levels. Between February 2020 and 2022, Children’s Hospital and Medical Center reported that the share of children aged 11 and older with positive depression screenings rose from about 15% to over 20%, reaching nearly 30% in some months, surpassing national averages of approximately 15% (27). Consistent with national trends during the COVID-19 pandemic, Nebraska experienced a marked increase in youth mental health crises during this period. From 2020 to 2021, the rate rose from approximately 15 to 22%, and from 2021 to 2022 it increased further to nearly 30%. Calls to Omaha’s Boys Town National Hotline related to child anxiety also increased by more than 50% during the same period, signaling heightened psychological distress (27).

**Figure 2:**
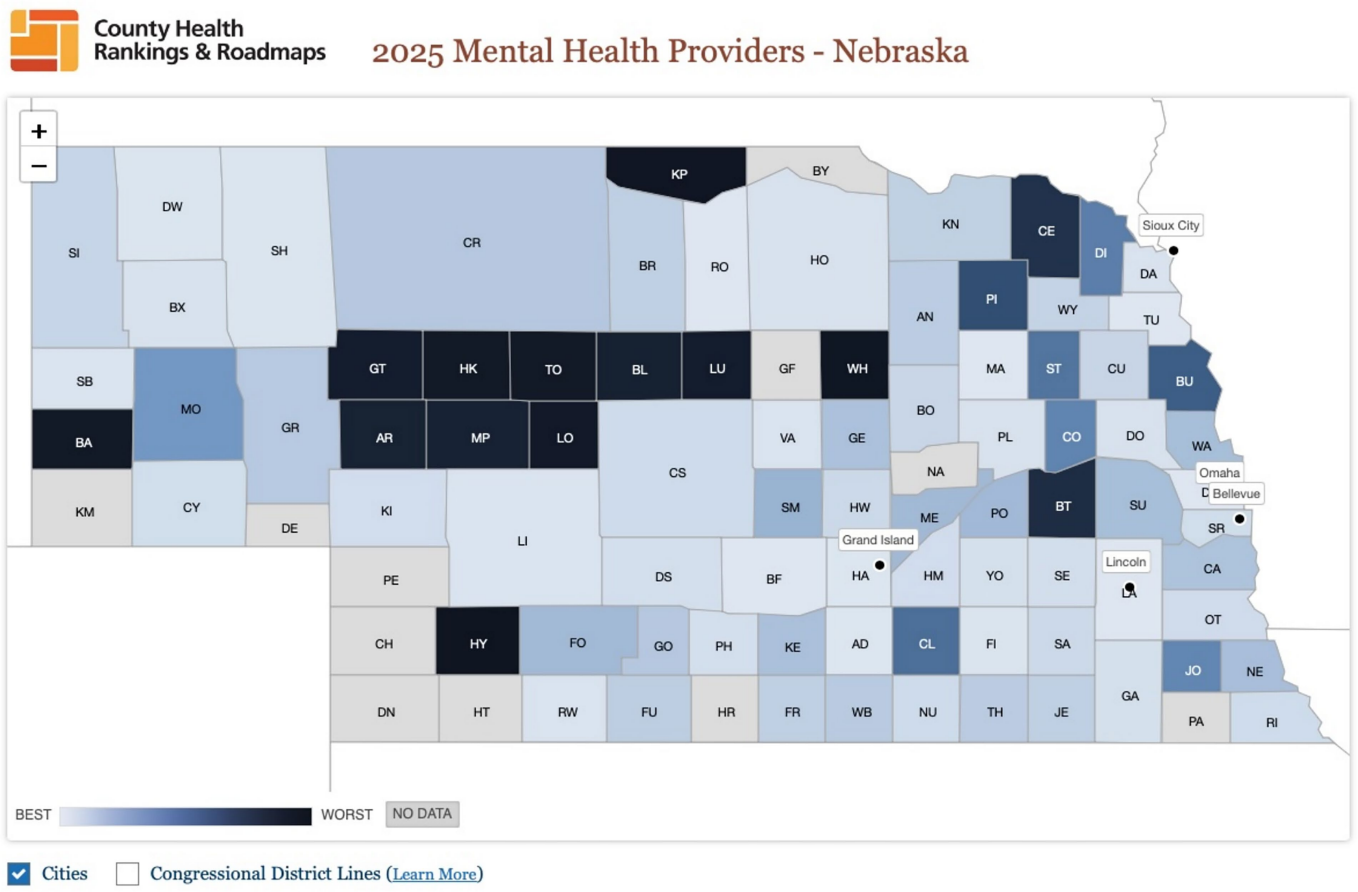
Geographic distribution of mental health providers in Nebraska, 2025 (14).

Workforce shortages compound these challenges: 88 of Nebraska’s 93 counties are designated as behavioral health shortage areas, and 29 counties have no practicing mental health providers at all. Only 26% of licensed behavioral health providers serve rural areas, further limiting access for children outside urban centers (10). According to the *County Health Rankings & Roadmaps 2025* and the Nebraska Pediatric Mental Healthcare Access Partnership, rural areas have just three psychiatrists per 100,000 residents compared to 11.8 in urban areas, and 8.7 psychologists per 100,000 residents compared to 27.9 in urban areas (28, 29). Figure 2 below illustrates the geographic distribution of mental health providers across Nebraska in 2025, with darker shading indicating counties with fewer providers per capita (14). This visual highlights the pronounced disparities between rural and urban areas, underscoring the urgent need for targeted workforce expansion in underserved regions.

These conditions, escalating mental health concerns among youth and critical workforce gaps, underscore the need for targeted investments in pediatric mental health services, as emphasized by participants at the 2024 *Understanding Health Disparities in Nebraska* symposium. Their insights strongly support evidence-based, community-rooted solutions that meet children where they are: at school, at home, and online.

### 4.2 Integrated SDOH and mental health

Social determinants of health (SDOH), including access to housing, food security, and social supports, not only shape mental health outcomes but are also proactively improved by better mental health. For example, stable mental health can foster stronger school performance, community participation, and economic resilience. Integrated care models reflect this interplay. School-based comprehensive mental health systems that partner with food assistance or family resource programs have shown reductions in behavioral incidents and improved attendance and emotional well-being (30). Similarly, community health centers that combine behavioral health services with social service navigation, such as housing support or job training referrals, have reported better treatment adherence and patient satisfaction (31). Embedding mental health supports within broader SDOH strategies can therefore be a powerful, sustainable approach to improving outcomes in Nebraska and beyond. These integrated approaches, combining SDOH interventions with mental health support, demonstrate the potential for synergistic strategies that advance both objectives simultaneously.

### 4.3 Policy options for pediatric mental health services

To ensure impact and scalability, policy options should incorporate evidence-based programs (EBPs) with demonstrated effectiveness in improving youth mental health outcomes. Examples include school-based mental health training programs, integrated behavioral health models, and telehealth-enabled service delivery that addresses workforce shortages and access barriers. Increased funding for mental health services, focusing on expanding access to services, and reducing stigma are approaches supported by studies indicating that investments in school-based mental health programs and community outreach can significantly improve youth mental health outcomes and reduce suicide rates (32, 33).

- Increase state and federal funding

o Expand investment in pediatric mental health services.
o In 2022, Nebraska’s behavioral health budget was ~$307 million, with ~40% from federal sources (34).
o Rural areas have only 8.7 psychologists and 3 psychiatrists per 100,000 residents, compared to 27.9 psychologists and 11.8 psychiatrists in urban regions (35).
o About 20.2% of children are diagnosed with behavioral conditions, but only 62.4% receive counseling (36).
o Prioritize hiring and training licensed independent mental health practitioners (LIMHPs) and school-based providers in underserved areas.
o Access is further constrained by insurance coverage limitations. Many plans still impose annual session caps, often ranging from 12 to 20, which are frequently insufficient for children with complex needs (37). Despite parity laws, reimbursement rates remain low in many areas, especially under Medicaid, where provider payments in Nebraska have not kept pace with Medicare and have been further complicated by recent policy changes that reduced total compensation for some patients (38). Waitlists add yet another layer of delay. Many families, especially in rural areas, face multi-month waits before accessing pediatric mental health care (39). These systemic barriers affirm that increased funding must be paired with strategies to expand provider capacity, enhance insurance coverage, and accelerate access.

- Enhance community outreach programs

o Collaborate with schools, community centers, and clinics to offer early-intervention programs.
o Focus on awareness, stigma reduction, and connecting families to services.

- Expand telehealth services

o Increase broadband access and insurance coverage for virtual mental health care.
o Support delivery of services like telepsychiatry and remote therapy.
o Integrate 988 crisis services into telehealth platforms. Ensure that telehealth providers, school-based mental health programs, and mobile health units are equipped to connect patients in crisis directly with the 988 Suicide and Crisis Lifeline. This integration leverages existing resources and can streamline crisis intervention, particularly in rural areas with limited in-person mental health resources.

- Strengthen school-based mental health programs

o Implement in-school counseling and mental health education.
o Partner with educational systems to integrate services into curricula and student supports.
o Incorporate evidence-based programs such as *Youth Mental Health First Aid* training for teachers, school counselors, and other school personnel to better equip them to recognize the signs of mental health crises, respond appropriately, and connect students to needed resources (40).
o Additionally, schools can and should facilitate access to telehealth services. First, by ensuring broadband infrastructure is sufficient and providing a community resource that is accessible. Second, by designating private spaces where students can participate confidentially in virtual mental health sessions, schools can support access and promote participation in mental health care (41–44). Such models have been successfully implemented in school-based health centers nationwide, demonstrating improved access to care, reduced stigma, and better coordination between educational and healthcare systems.

- Host community events and public awareness campaigns

- o Organize events like mental health fairs, speaker series, and media campaigns, which are demonstrated to improve health care usage and outcomes, especially among younger people (45).
- o Normalize mental health conversations through schools, libraries, churches, and local media.

### 4.4 Implications

Improving mental health services will enhance mental health outcomes for children, reduce rates of suicide, development of substance use disorders, and mental health crises, and provide long-term benefits for physical, behavioral, and social health (8, 25). For instance, studies show that early intervention in youth mental health issues can prevent the development of more severe conditions, leading to better overall health outcomes (32, 33).

## 5 Social determinants of health

### 5.1 Current situation

Social determinants of health (SDOH) are non-medical factors that influence health outcomes. According to Healthy People 2030, they encompass the conditions in which people are born, grow, live, work, and age, including socioeconomic status, environment, and education (46). These factors significantly influence health outcomes, accounting for up to 80% of overall health status in the United States (24, 47). In Nebraska, disparities are evident across regions. For example, East Omaha experiences concentrated socioeconomic deprivation, leading to poorer health outcomes compared to more affluent areas like West Omaha (48).

In addition to structural disparities, systemic racism and discrimination within Nebraska’s healthcare settings further deepen health inequities. Black, Hispanic, Indigenous, and immigrant populations often encounter implicit bias, language barriers, and limited access to culturally and linguistically appropriate care; factors that contribute to mistrust in medical institutions and inadequate treatment. For example, Yazidi and Sudanese refugee communities in Lincoln face significant barriers related to health literacy and navigation of the healthcare system (20). These inequities are reflected in statewide outcomes: Nebraska’s Black infant mortality rate (11.6 per 1,000 live births) is more than double that of all other racial groups, and obesity and delayed prenatal care rates are significantly higher among Black and American Indian populations (49), underscoring the urgent need to address social and systemic inequities as part of the state’s health equity agenda.

Both obesity and delayed prenatal care in Nebraska are influenced by a complex set of interrelated barriers that vary across communities. Limited access to fresh, nutritious foods remains a significant challenge. Many food banks and pantries, particularly in rural areas and urban food deserts, primarily distribute shelf-stable, processed items, reducing opportunities for healthy eating (18). Prenatal care access is hindered by a shortage of obstetricians/gynecologists in rural counties, many of which have no local providers, forcing patients to travel long distances or forgo care entirely (10–12). Even when services are available, insurance limitations and restrictive eligibility criteria for support programs prevent participation, and inadequate broadband infrastructure can make participation in telehealth and health education difficult. These barriers often intersect, amplifying inequities for low-income families and historically underserved populations. Addressing them requires not only targeted new policies but also a critical review of existing programs and regulations to ensure they do not inadvertently perpetuate disparities, thereby creating solutions that are both effective and sustainable.

### 5.2 Policy options for addressing social determinants of health

Implement community-based interventions targeting SDOH, including educational programs, housing initiatives, and food security measures. In addition, addressing health disparities requires coordinated solutions that meet families’ interconnected needs. For example, access to affordable childcare can enable workforce participation, while financial counseling, when paired with policies ensuring livable wages, can help families maximize available resources without placing undue burden on those already working multiple jobs.

- Educational programs: Fund accessible programs on healthy living, nutrition, disease prevention, and health literacy. Build on models like the *Center for People in Need* in Lincoln and California’s *Health Navigator Program* to expand statewide efforts (50).
- Affordable housing initiatives: Invest in safe, affordable housing through partnerships with local governments and housing authorities.
- Food security programs: Expand food banks, community gardens, and nutrition workshops. Support initiatives like teaching kitchens and DIY gardens.
- Transportation services: Improve public transit and offer transportation assistance, especially in rural and underserved areas.
- Collaborative community partnerships: Build cross-sector networks to provide wraparound services, integrating healthcare, social services, and community organizations to offer employment support, financial counseling, and childcare assistance. Such comprehensive approaches can help address multiple social determinants of health simultaneously, improving access to resources and supporting long-term community well-being.

### 5.3 Implications

Addressing social determinants will reduce health disparities, enhance quality of life, and improve overall health outcomes for underserved populations. Initiatives such as improving housing conditions and increasing access to nutritious foods have been shown to significantly improve health outcomes in various communities (51).

## 6 Healthcare access for underserved populations

### 6.1 Current situation

Underserved populations face significant barriers to healthcare access, including a lack of insurance, transportation issues, and limited availability of services. Nationally, rural Americans face similar challenges, with about 20% living in Health Professional Shortage Areas (12, 52). In Nebraska, many individuals in rural areas rely on emergency departments for primary care due to these barriers (8). This unbalanced use of emergency departments for non-emergency needs leads to negative outcomes for individuals, such as fragmented care, delayed diagnoses, and higher out-of-pocket costs (53). It also overburdens emergency departments, reducing their capacity to respond to true emergencies and straining healthcare resources across the community (54).

### 6.2 Policy options for expanding healthcare access in underserved populations

To strengthen Nebraska’s public health infrastructure, the state should expand the number and geographic reach of community health centers in underserved areas. Increasing access to affordable insurance coverage and providing enrollment assistance will help individuals overcome financial barriers to care. Enhancing non-emergency medical transportation services, particularly in rural regions, is essential for ensuring consistent access to routine and preventive healthcare. Finally, all healthcare settings should offer culturally and linguistically appropriate services to better meet the needs of Nebraska’s diverse population.

Recent changes to Medicaid eligibility and funding could have particularly severe consequences for Nebraska’s rural hospitals. Critical access facilities, which serve as the only hospital within large geographic areas, operate on narrow fiscal margins and rely heavily on Medicaid reimbursements. As Nebraska Rep. Mike Flood answered questions regarding H. R. 1, also known as the “One Big Beautiful Bill Act,” during an Augugst 4^th^, 2025 public townhall, it became clear that shifting costs to these facilities will increase uncompensated care burdens, jeopardizing their viability (55). This risk is compounded by provider shortages and aging infrastructure in rural communities. National analyses similarly warn that Medicaid cuts may accelerate rural hospital closures, further limiting access to primary and emergency care (56–60). To sustain access, policies should include targeted rural hospital stabilization funds, safeguards for Medicaid expansion, and recruitment incentives to attract providers to underserved areas.

- Expand community health centers

o Increase the number of centers in rural and underserved areas.
o Ensure availability of preventive, primary, and mental health services.

Reference relevant legislation supporting statewide resource distribution; assess current implementation and gaps.

- Invest in mobile health units: cross-sector tie-in

o Invest in Mobile Health Units through cross-sector partnerships.
o Collaborate with public library bookmobiles, Meals on Wheels, syringe exchange programs, and other community-based services to deliver healthcare, screenings, and resources directly to underserved populations.
o These partnerships can extend reach and reduce barriers for hard-to-access communities. Oregon’s MHU Program is an exemplar model (61).

- Implement transportation assistance programs

o Provide free or subsidized rides to healthcare appointments.
o Collaborate with local transit providers.
o Example: Existing Lincoln-based services could serve as local models.

- Ensure language accessibility

- o Require translation, interpretation, and captioning services in all healthcare settings.
- o Offer forms and materials in multiple languages.
- o Avoid reliance on family members, especially children, for translation (62).

- Expand telehealth services

o Improve broadband access in underserved regions.
o Support ongoing federal and local broadband expansion programs.
o Provide training for providers in telehealth delivery.
o Encourage leadership from universities, especially land-grant institutions, on telehealth access and workforce development.
o Expand Telehealth Services across diverse delivery channels. Build on school-based telehealth infrastructure and incorporate Employee Assistance Program (EAP) telehealth offerings for working populations.
o Explore policies to ensure cell phone calls, texting, and video visits for telehealth are free for patients and do not count against data or minutes, thereby removing cost-related barriers to virtual care.

### 6.3 Implications

Enhancing healthcare access will increase utilization of preventive and primary care, reduce reliance on emergency services, and improve health outcomes for underserved communities. While increasing the number of community health centers in rural areas remains an important goal (15, 52), feasibility must be assessed in light of current Medicaid changes and the risk of rural hospital closures. Strategic planning should prioritize sustaining existing facilities and programs and integrating them into broader telehealth and mobile health strategies.

## 7 Actionable recommendations

**Figure 3:**
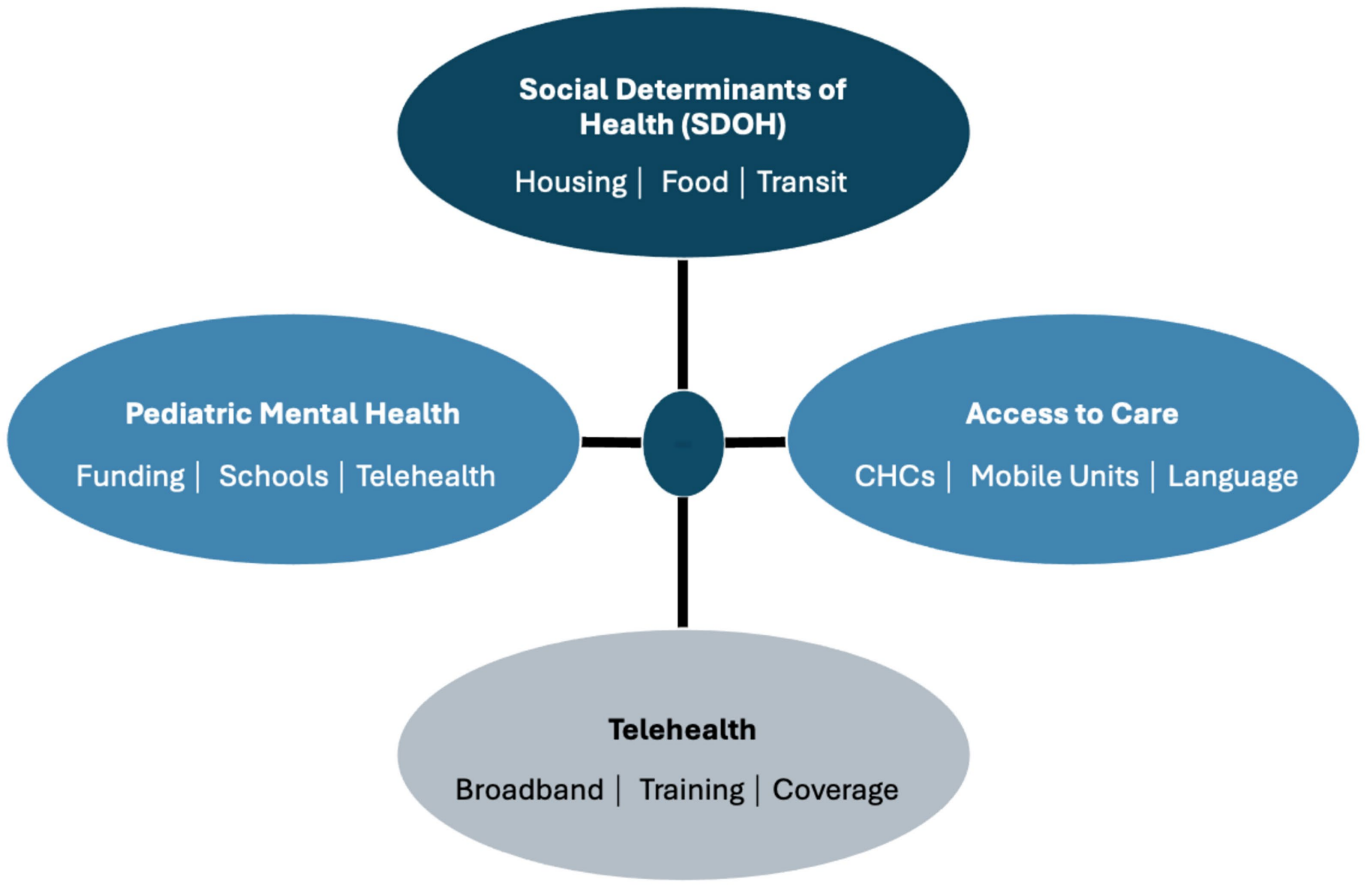
Interconnected strategies and actionable recommendations.

To meaningfully reduce health disparities in Nebraska, it is essential to recognize how core policy strategies intersect across multiple domains. Figure 3 illustrates how key actionable recommendations, focusing on pediatric mental health, access to care, telehealth, and social determinants of health (SDOH), are interconnected and collectively drive progress toward health equity. Rather than addressing these areas in isolation, coordinated action can produce broader, more sustainable outcomes.

- Increase funding for mental health services

o Allocate state and federal resources for child and adolescent mental health care, including expanding the workforce of school-based mental health providers.
o Support the implementation of evidence-based programs such as *Youth Mental Health First Aid* to equip educators and staff in identifying and responding to youth mental health needs.
o Prioritize hiring and training providers in rural and underserved areas.
o This strategy improves youth outcomes (8, 54).

- Implement community-based SDOH interventions

o Partner with local organizations to address housing, education, and food security.
o Collaborate with schools, clinics, and community centers to build support networks.
o Community-based approaches reduce the health impacts of social determinants (15, 51).

- Enhance healthcare access

- o Expand community health centers and mobile units in underserved areas.
- o Offer transportation assistance and language services.
- o Promote telehealth to fill service gaps and reduce emergency care reliance (15, 52).

- Education and awareness campaigns

o Launch statewide efforts to reduce stigma and promote mental health resources.
o Educate communities about SDOH and preventive care.
o Awareness campaigns improve help-seeking behaviors and service use (8, 15).

- Policy and legislative advocacy

o Support legislation that funds mental health, SDOH initiatives, and care access.
o Engage a broad coalition of stakeholders, including policymakers, healthcare providers, community leaders, school personnel, and families, to ensure that proposed policies are responsive to community needs and priorities.
o Conduct a systematic review of current local, state, and federal policies to identify those that may contribute to or exacerbate health disparities, and revise or replace them with equitable alternatives.
o Legislative action is key to long-term, sustainable change (51, 52).

## 8 Ethical considerations

Expanding interventions in mental health and social determinants of health must be undertaken with a commitment to ethical principles. Protecting privacy, particularly in telehealth and school-based mental health services, requires compliance with the Health Insurance Portability and Accountability Act (HIPAA) and the Family Educational Rights and Privacy Act (FERPA), and other relevant regulations, alongside transparent consent processes that are age-appropriate and culturally sensitive. Policy design should ensure equitable access so that rural, low-income, and historically marginalized communities benefit from new services, rather than face additional barriers. Incorporating community input into program development can help address cultural and linguistic needs, avoid stigmatization, and build trust between providers and residents. These ethical safeguards are essential for ensuring that interventions are both effective and socially responsible.

## 9 Implementation challenges and considerations

While the recommendations outlined in this policy brief are grounded in evidence, their implementation will require careful navigation of practical barriers. Rural and economically disadvantaged communities in Nebraska face unique constraints, including persistent healthcare workforce shortages, limited funding streams, and political challenges that may delay or limit policy adoption. For example, sustaining mobile health units or expanding school-based mental health programs may require stable funding beyond short-term grants, while telehealth expansion is contingent on adequate broadband infrastructure. Workforce pipeline programs, such as loan repayment incentives for rural providers, can help address shortages, but these require legislative support and budget allocations. Similarly, building bipartisan coalitions and engaging community stakeholders early in the policy development process can mitigate political resistance and improve feasibility. Positively, however, several of the policy recommendations presented here have cross-sectional benefit e.g., expanding telehealth infrastructure in schools helps increase access in rural areas where providers are scarce, provide access to different provider types, and support pediatric mental health. Similarly, using existing infrastructure to support mobile clinics can provide general care, be mobilized for acute responses, and adapted to new and/or emerging concerns. However, all approaches require support. Integrating implementation strategies alongside policy design will be critical to ensure these recommendations are not only adopted but also sustained over time.

## 10 Conclusion

Addressing health disparities in Nebraska requires a multifaceted approach that includes enhancing mental health services, addressing social determinants of health, and improving healthcare access for underserved populations. By implementing targeted policies and actionable recommendations, Nebraska can make significant strides toward achieving health equity and improving the well-being of all its residents. These efforts can serve as a model for other states facing similar health disparities, demonstrating the potential for targeted interventions to create equitable health outcomes nationwide. Framing our recommendations within the Social Ecological Model underscores the need for coordinated action across policy, community, interpersonal, and individual levels. This multi-layered approach not only addresses immediate gaps in care and services but also builds the structural conditions necessary for long-term, equitable health outcomes (3–5). Future research could extend this work by conducting a systematic review or meta-analysis to evaluate the quality, representativeness, and methodological strengths and limitations of the evidence base for health disparity policy interventions in Nebraska and comparable states.

## References

[1] Centers for Disease Control and Prevention. *Health disparities*. (2022). Available online at: https://www.cdc.gov/healthyyouth/disparities/index.htm

[2] Parkinson E Hill T. *Legislative summary: Addressing disparities in access to health care*. Washington, DC: National Conference of State Legislatures (2023). Available online at: https://www.ncsl.org/state-legislatures-news/details/legislative-summary-addressing-disparities-in-access-to-health-care.

[3] Bronfenbrenner U. The ecology of human development: Experiments by nature and design. Cambridge, MA: Harvard University Press (1979).

[4] McLeroy KR Bibeau D Steckler A Glanz K. An ecological perspective on health promotion programs. Health Educ Q. (1988) 15:351–77. doi: 10.1177/109019818801500401, PMID: 3068205

[5] Golden SD Earp JAL. Social ecological approaches to individuals and their contexts: twenty years of health education & behavior health promotion interventions. Health Educ Behav. (2012) 39:364–72. doi: 10.1177/1090198111418634, PMID: 22267868

[6] National Center for Health Statistics. Health, United States, 2020–2021: Annual perspective. Hyattsville, MD: National Center for Health Statistics (2023). 36888733

[7] Bashford G Cantu-Hines M Crawford L George R Hughes M Yan C. *Nebraska’s health divide: Insights from University of Nebraska-Lincoln Grand challenges health equity symposium 1*. Lincoln, NE: University of Nebraska-Lincoln. (2024). Available online at: https://cms.unl.edu/journalism/health-disparities/sites/unl.edu.journalism.health-disparities/files/media/file/Symposium%201%20White%20Paper.pdf.

[8] Tsai E Allen P Saliba LF Brownson RC. The power of partnerships: state public health department multisector collaborations in major chronic disease programme areas in the United States. Health Res Policy Syst. (2022) 20:80. doi: 10.1186/s12961-021-00765-3, PMID: 35804420 PMC9264297

[9] National Conference of State Legislatures. *Improving rural health: State policy options for increasing access to care*. Washington, DC (2020). Available online at: https://www.ncsl.org/health/improving-rural-health.

[10] Tak HJ Chakraborty B Carritt N Palm DW Deras M Horner RD. *The state of the Nebraska healthcare workforce: 2022 update*. Omaha, NE: UNMC Center for Health Policy (2022). Available online at: https://www.unmc.edu/rural-health/_documents/healthcare_workforce_status_2022.pdf.

[11] Nebraska Hospital Association. *Roadmap to strong rural health care*. (2024). Available online at: https://www.nebraskahospitals.org/advocacy/roadmap-to-strong-rural-health-care.html.

[12] Warshaw R. *Health disparities affect millions in rural U.S. communities*. Association of American Medical Colleges. (2017). Available online at: https://www.aamc.org/news/health-disparities-affect-millions-rural-us-communities.

[13] BroadbandNow. *Nebraska internet coverage & availability in 2024*. (2024). Available online at: https://broadbandnow.com/Nebraska.

[14] University of Wisconsin Population Health Institute. *County Health Rankings and Roadmaps 2025. University of Wisconsin School of Medicine and Public Health*. (2025). Available online at: https://www.countyhealthrankings.org.

[15] Agency for Healthcare Research and Quality. *2021 National Healthcare Quality and disparities report. U.S*. Department of Health and Human Services (2021). Available online at: https://www.ahrq.gov/research/findings/nhqrdr/nhqdr21/index.html.

[16] U.S. Census Bureau. *QuickFacts*. Nebraska: Omaha City, Nebraska. (2023). Available online at: https://www.census.gov/quickfacts/fact/table/NE,omahacitynebraska.

[17] United Health Foundation. *America’s health rankings analysis of U.S. Census Bureau, American community survey, 1-year dataset*. (2023). Available online at: https://www.americashealthrankings.org/explore/measures/pct_rural_b/NE.

[18] Douglas County Health Department. *Healthy food for all: Community health needs and solutions plan*. (2021). Available online at: https://www.douglascountyhealth.com/images/CHNS/Health_Food_4_All/Healthy_Food_For_All_Full_Plan.pdf.

[19] U.S. Census Bureau. *QuickFacts*. Lancaster County, Nebraska. (2020). Available online at: https://www.census.gov/quickfacts/lancastercountynebraska.

[20] Bonderson A. *UNL study shows barriers to healthcare for Yazidi refugees in Nebraska. Nebraska public media*. (2022). Available online at: https://nebraskapublicmedia.org/en/news/news-articles/unl-study-shows-barriers-to-healthcare-for-yazidi-refugees-in-nebraska/.

[21] Nath R Matthews D Hobaica S Eden TM Taylor AB DeChants JP. *2024 U.S. National Survey on the mental health of LGBTQ+ young people by state*. West Hollywood, CA: The Trevor Project (2025). Available online at: www.thetrevorproject.org/survey-2024-by-state.

[22] Rothstein R. The color of law: A forgotten history of how our government segregated America. New York: Liveright Publishing (2017). 243 p.

[23] Gerken M Batko S Fallon K Fernandez E Williams A Chen B. *Addressing the legacies of historical redlining: Correlations with measures of modern housing instability*. Urban Institute (2023). Available online at: https://www.urban.org/research/publication/addressing-legacies-historical-redlining (Accessed August 8, 2025).

[24] Hood CM Gennuso KP Swain GR Catlin BB. County health rankings: relationships between determinant factors and health outcomes. Am J Prev Med. (2016) 50:129–35. doi: 10.1016/j.amepre.2015.08.024, PMID: 26526164

[25] U.S. Census Bureau. *QuickFacts*. Nebraska. (2023). Available online at: https://www.census.gov/quickfacts/NE.

[26] Ehrenberg C Ramírez JP Kubiak N. *State health assessment Nebraska Department of Health and Human Services* (2023).

[27] The Reader. *The kids Aren’t OK: Mental health issues skyrocketed for Nebraska children during pandemic*. (2022). Available online at: https://thereader.com/2022/04/15/the-kids-arent-ok-mental-health-issues-skyrocketed-for-nebraska-children-during-pandemic/.

[28] University of Wisconsin Population Health Institute. *County Health Rankings and Roadmaps 2025: Mental health providers*. (2025). Available online at: https://www.countyhealthrankings.org/health-data/community-conditions/health-infrastructure/clinical-care/mental-health-providers.

[29] Nebraska Department of Health and Human Services. *Nebraska Partnership for Mental Healthcare Access in pediatrics (NEP-MAP)*. (2025). Available online at: https://dhhs.ne.gov/Pages/Nebraska-Pediatric-Mental-Healthcare-Access-Partnership.aspx.

[30] Hoover S Lever N Sachdev N Bravo N Schlitt J Acosta Price O. *Advancing comprehensive school mental health: Guidance from the field*. Baltimore, MD: National Center for School Mental Health. University of Maryland School of Medicine (2019). Available online at: www.schoolmentalhealth.org/AdvancingCSMHS.

[31] Castillo EG Ijadi-Maghsoodi R Shadravan S Moore E Mensah MO Docherty M. Community interventions to promote mental health and social equity. Curr Psychiatry Rep. (2019) 21:35. doi: 10.1007/s11920-019-1017-0, PMID: 30927093 PMC6440941

[32] Bruns EJ Walker JS Bernstein A Daleiden E Pullmann MD Chorpita BF. Family voice with informed choice: coordinating wraparound with research-based treatment for children and adolescents. J Clin Child Adolesc Psychol. (2014) 43:256–69. doi: 10.1080/15374416.2013.859081, PMID: 24325146 PMC3954919

[33] Hoagwood KE Olin SS Horwitz S McKay M Cleek A Gleacher A. Scaling up evidence-based practices for children and families in New York state: toward evidence-based policies on implementation for state mental health systems. J Clin Child Adolesc Psychol. (2014) 43:145–57. doi: 10.1080/15374416.2013.869749, PMID: 24460518 PMC3954943

[34] Nebraska Department of Health and Human Services. *State budget and expenditure reports*. (2022). Available online at: https://dhhs.ne.gov

[35] Nebraska Department of Health and Human Services. *Nebraska Partnership for Mental Healthcare Access in pediatrics*. (2024). Available online at: https://dhhs.ne.gov/Pages/Nebraska-Pediatric-Mental-Healthcare-Access-Partnership.aspx.

[36] Voices for Children in Nebraska. *Kids count in Nebraska 2022 data report*. (2022). Available online at: https://voicesforchildren.com/wp-content/uploads/2023/05/Final_KidsCount2022_.pdf.

[37] Gouraud R. *Does insurance cover therapy? A complete guide to mental health coverage*. (2025). Available online at: https://www.therapyden.com/blog/insurance-cover-therapy-guide.

[38] Dunker C. *Nebraska mental health care providers say changes put “poorest of poor” at-risk*. Lincoln Journal Star (2024). Available online at: https://insurancenewsnet.com/oarticle/nebraska-mental-health-care-providers-say-changes-put-poorest-of-poor-at-risk-2.

[39] Su D Karsting K Ern J Quinn A Walker C. *Provider perspectives of mental health needs and services among children in Nebraska*. University of Nebraska Medical Center (2021).

[40] National Council for Mental Wellbeing. *Mental health first aid for youth*. (2024). Available online at: https://www.mentalhealthfirstaid.org/population-focused-modules/youth/.

[41] Stephan S Lever N Bernstein L Edwards S Pruitt D. Telemental health in schools. J Child Adolesc Psychopharmacol. (2016) 26:266–72. doi: 10.1089/cap.2015.0019, PMID: 26982886

[42] Love H Panchal N Schlitt J Behr C Soleimanpour S. The use of telehealth in school-based health centers. Glob Pediatr Health. (2019) 6:2333794X19884194. doi: 10.1177/2333794X19884194, PMID: 31692723 PMC6811756

[43] U.S. Department of Education. *ConnectED initiative*. (2013). Available online at: https://obamawhitehouse.archives.gov/issues/education/k-12/connected.

[44] Jancsura M Bowers K Telfer N Holm-Hansen C Arthur M Rosenfeld L. *Improving access to health care: The challenges and potential of Telehealth and Telementoring*. Evidence-to-Impact Collaborative, Penn State University (2023). Available online at: https://evidence2impact.psu.edu/resources/improving-access-to-health-care-the-challenges-potential-of-telehealth-telementoring/.

[45] Tam MT Wu JM Zhang CC Pawliuk C Robillard JM. A systematic review of the impacts of media mental health awareness campaigns on young people. Health Promot Pract. (2024) 25:907–20. doi: 10.1177/15248399241232646, PMID: 38468568 PMC11370183

[46] Healthy People 2030, U.S. Department of Health and Human Services, Office of Disease Prevention and Health Promotion. *Social determinants of health*. Available online at: https://odphp.health.gov/healthypeople/priority-areas/social-determinants-health. (Accessed August 10, 2025).

[47] Magnan S. Social determinants of health 101 for health care: five plus five. Washington, DC: National Academy of Medicine (2017).

[48] United Way of the Midlands. *Community profile: A vibrant place to call home*. Available online at: https://unitedwaymidlands.org/signals-and-trends-report/community-profile/ (Accessed Janaury 19, 2025)

[49] Nebraska Department of Health and Human Services. *Office of Health Disparities and Health Equity*. Annual Report. (2023). Available online at: https://dhhs.ne.gov/Reports/Office-of-Health-Disparities-Annual-Report-2023.pdf.

[50] DeMilto L Nakashian M. In M. McKaughan, Ed. Using social determinants of health data to improve health care and health: A learning report. Robert Wood Johnson Foundation. (2016). Available at: https://housingis.org/sites/default/files/RWJF-SDOH-Learning-Report.pdf

[51] United Health Foundation. *2021 health disparities report*. (2021). Available online at: https://assets.americashealthrankings.org/app/uploads/2021_ahr_health-disparities-comprehensive-report_final.pdf.

[52] Colizzi M Lasalvia A Ruggeri M. Prevention and early intervention in youth mental health: is it time for a multidisciplinary and trans-diagnostic model for care? Int J Ment Health Syst. (2020) 14:23. doi: 10.1186/s13033-020-00356-9, PMID: 32226481 PMC7092613

[53] Agency for Healthcare Research and Quality (AHRQ). *National Healthcare Quality and disparities report. U.S*. Department of Health and Human Services (2023). Available online at: https://www.ahrq.gov/research/findings/nhqrdr/nhqdr23/index.html.

[54] Centers for Disease Control and Prevention (CDC). *Improving access to children’s mental health care*. (2023). Available online at: https://www.cdc.gov/childrensmentalhealth/access.html

[55] Nebraska Public Media. Nebraska 1st district rep. Mike Flood Townhall meeting on HR 1 healthcare. (2025). Available online at: https://www.youtube.com/watch?v=zleBod9OszY

[56] American Hospital Association. *Rural hospitals at risk: Cuts to Medicaid would further threaten access*. American Hospital Association (2025). Available online at: https://www.aha.org/fact-sheets/2025-06-13-rural-hospitals-risk-cuts-medicaid-would-further-threaten-access.

[57] Sheffert E Sandalow M. *Rural hospitals and the rural health transformation program: What comes next?* Bipartisan Policy Center (2025). Available online at: https://bipartisanpolicy.org/blog/rural-hospitals-rural-health-transformation-program-what-comes-next/.

[58] National Rural Health Association. *Statement on Medicaid changes and rural hospital viability*. (2025). Available online at: https://www.ruralhealth.us.

[59] Euhus R Williams E Burns A Rudowitz R. *Allocating CBO’S estimates of Federal Medicaid Spending Reductions across the states: Enacted reconciliation package*. Kaiser Family Foundation (2025). Available online at: https://www.kff.org/medicaid/issue-brief/allocating-cbos-estimates-of-federal-medicaid-spending-reductions-across-the-states-enacted-reconciliation-package/.

[60] Glenza J Chidi G. *Trump’s Medicaid cuts are coming for rural Americans: ‘it’s going to have to hit them first.’* Guardian (2025). Available online at: https://www.theguardian.com/us-news/2025/jul/04/rural-americans-medicaid-cuts-trump-bill.

[61] Oregon Health Authority. *Mobile health units program overview*. (2022). Available online at: https://www.oregon.gov/oha/hsd/pages/mhu-program.aspx

[62] National Academies of Sciences, Engineering, and Medicine. Communicating clearly about health care: Recommendations to improve patient understanding and support informed decision making. Washington, DC: The National Academies Press (2017).

